# Diffuse tensor cardiac MRI evaluation of fiber architecture of athlete hypertrophic heart in vivo

**DOI:** 10.1186/1532-429X-14-S1-P170

**Published:** 2012-02-01

**Authors:** Ming-Ting Wu, Wen-Yih I Tseng, Mao-Yuan M Su, Van J Weeden, Timothy G Reese

**Affiliations:** 1 Department of Radiology, National Yang Ming University, Taipei, Taiwan; 2 Center for Optoelectronic Biomedicine, National Taiwan University, Taipei, Taiwan; 3 Department of Medical Imaging, National Taiwan University Hospital, Taipei, Taiwan; 4 National Yang Ming University, Taipei, Taiwan

## Background

The fiber architecture adaption in physiological hypertrophy of the althete heart is still delusive. We aimed to use diffusion tensor cardiac MR (DT-CMR) to evaluate the tissue property and fiber architecture of elite athlete heart.

## Methods

Eight elite athletes of Marathon runner (endurance-training type), 8 of weight-lifter (strength-training type) and 8 ordinary style (medical interns) were enrolled. Each subject received a CMR study on a 1.5 T scanner including 1. cine SSPF of a stack of LV short axis for LV mass and function; 2 DT-CMR, ECG-gated stimulated echo diffuse EPI on three levels of LV. Diffuse tensor composed of 6 directions and b value = 300 mm2/sec. 3. phase-contrast flow measurement at ascending aorta for stroke volume. The data were compared between groups and correlated between the parameters.

## Results

The myocardium showed no difference of mean diffusivity (MD) and fractional anisotropy between the groups. Weight lifter showed increase of stroke volume / BSA and LV mass / BSA as compared to runner and ordinary groups. The fiber architecture showed an increased proportion of right-handed helical fibers (mainly in the subendocardial zone) in runner and lifter equally, as compared to ordinary group. Putting all 24 subjects together, there was a linear regression between the proportion of right-handed helical fiber and LV mass (R square = 0.38, p = 0.002).

## Conclusions

DT-CMR revealed the physiological hypertrophy of athlete heart was mainly due to right-handed helical fibers. This underscores the important role of subendocardial fiber on the LV function.

## Funding

N/A

**Figure 1:**
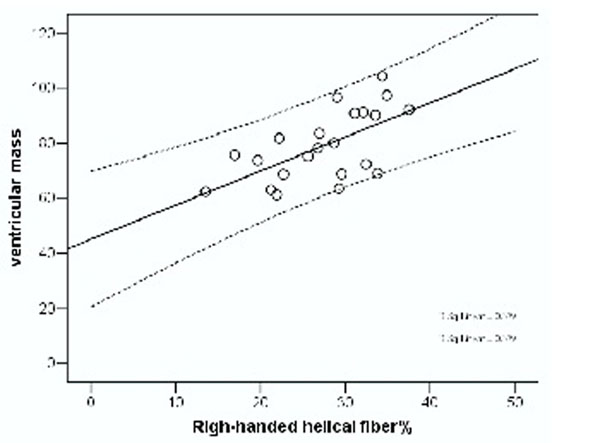
Regression between LV mass and right-handed helical fiber percentage across the 24 subjects.

